# Molecular Subtypes and Prognostic Signature of Pyroptosis-Related lncRNAs in Glioma Patients

**DOI:** 10.3389/fonc.2022.779168

**Published:** 2022-02-14

**Authors:** Guilong Tanzhu, Na Li, Zhanzhan Li, Rongrong Zhou, Liangfang Shen

**Affiliations:** ^1^ Department of Oncology, Xiangya Hospital, Central South University, Changsha, China; ^2^ National Clinical Research Center for Geriatric Disorders, Xiangya Hospital, Central South University, Changsha, China

**Keywords:** glioma, lncRNA, pyroptosis, prognosis model, bioinformation analysis

## Abstract

The relationship between pyroptosis-related long non-coding RNAs (pyroptosis-related lncRNAs) and glioma prognosis have not been studied clearly. Basing on The Cancer Genome Atlas and The Chinese Glioma Genome Atlas datasets, we firstly identified 23 pyroptosis-related lncRNAs with Pearson coefficient |r| > 0.5 and p < 0.001. The survival probability was lower in cluster 1. 13 lncRNAs was included into signature and divided all the glioma patients into two groups, among which survival probability of the high-risk group was lower than that in low-risk group (P<0.001). The risk score was higher in the age>60, dead grade 3, cluster 1 and immune score high groups. Furthermore, subgroup analysis showed patients with different grades, IDH and 1p19ql state distinguished by the median of risk score had different survival probability. Risk score was one of independent factors for glioma prognosis, and 1-, 3-, 5-years survival were calculated in nomogram. Meanwhile, the same as the median risk score in TCGA cohort, the glioma patients from CGGA were categorized into two groups and validated the outcome mentioned above(P<0.01). GO and KEGG analysis revealed the immunity process of the targeted genes. Thus, the immune filtration we compared showed naive B cell, resting dendritic cells, activated NK cells, activated Mast cells, monocytes are higher in low-risk group. Moreover, level of the activated NK cells, M0-and M1 Macrophages was in positive relationship with the risk score. Besides, competing endogenous RNA (ceRNA) network display interaction among microRNA, lncRNAs and their targeted genes. Pyroptosis-related lncRNAs could be a dependent prognosis factor and maybe linked to the immune response in glioma. This prognosis signature had potential value in estimate the survival of the patients with glioma.

## Introduction

Gliomas, which is one of the most common types of primary central nervous malignant tumors, has undesirable prognosis (1). Based on the stage and clinical information, surgical resection, radiotherapy, and chemotherapy are the main therapies. However, the 5-year survival rate of glioma patients is less than 35% due to the lack of ideal and effective treatments methods (2). Gene expression features and related molecular signatures, such as isocitrate dehydrogenase genes 1 and 2 (IDH1/IDH2) mutant status, TERT promotor status, codeletion of chromosome arm 1p and 19q (1p/19q codel) play an important role in prognosis (3, 4). It’s possible that more comprehension into molecular feature will contribute to the diagnosis, prognosis, and treatment of glioma (5).

The term pyroptosis proposed by Cookson and Brennan in 2001,becoming the hotspot in recent years, is a new form of programmed cell death (PCD) (6). There are 33 relevant genes (7) modulating the pyroptosis process, and generating various effects on cancers. Previous studies have revealed the function in tumors. The formation of NLRP3 inflammatory corpuscles involve in the malignant transformation of lung bronchial epithelial cells (8, 9), and AIM2 promote proliferation and migration (10). In addition, as a type of procedural and inflammatory death, pyroptosis can inhibit tumor growth (11). Some vitro experiments on lung cancer showed pyroptosis was induced by the use of chemotherapy drugs such as dasatinib (12), paclitaxel and cisplatin (13, 14). A few relevant studies illustrated glioma and pyroptosis, which is mainly focus on the miRNA, circRNA and small molecule effect (15–19). Pyroptosis unfold the potential effects of becoming a novel therapeutic approach of cancer, and pyroptosis-related genes GSDMD can be a prognosis factor of NSCLC. It seems essential to construct pyroptosis-related genes prognosis signature to explore the genes functions, the tumor microenvironment and its relationship with immunotherapy.

Long noncoding RNAs (lncRNAs) have been identified as pivotal regulators in tumor, being as oncogenes or suppressors. Down-regulated lncRNAs MALAT1, TUG1 suppressed glioma cell growth and invasion, induced apoptosis as well. Furthermore, previous studies on lncRNAs indicated the therapeutic use of the future treatment in glioma (20). Apart from pyroptosis-related lncRNAs, some lncRNA related to immune, autophagy and N6-methylandenosin have been deeply searched in recent years (21–23). Thus, here we explore the function and the prognosis predictive capability of lncRNAs associated with pyroptosis in glioma patients.

## Materials and Methods

### Data Acquisition

We downloaded the RNA sequence data and clinical information of glioma from the The Cancer Genome Atlas (TCGA), which includes 59 normal human brain samples and 535 glioma samples. We obtained the validations dataset from the Chinese Glioma Genome Atlas (CGGA), including 1018 glioma patients’ mRNA expression profiles and clinical information. All the datasets from two datasets are normalized to fragment per kilobase million (FPKM) values. Patients without follow-up data or overall survival < 30 days were excluded. We got the lncRNAs annotation of TCGA and CGGA according to the genome references consortium human build 38 that can be downloaded from the GENCODE website. Finally, we identified 14086 lncRNAs from TCGA and 1360 lncRNAs from CGGA. 33 pyroptosis-related genes were extracted from the previous publications (6, 24–26).

### Identification and Cluster Analysis of Pyroptosis-Related lncRNAs

To identify the pyroptosis-related lncRNAs, we firstly performed the Pearson correlation analysis among pyroptosis-related genes and lncRNAs in TCGA and CGGA dataset. The “p” package was applied to find out the pyroptosis-related lncRNAs in each dataset with a screening condition that Pearson coefficient |r| > 0.5 and P < 0.001. Univariate COX regression was applied to identify the prognosis-related lncRNAs in two datasets (P<0.001). Then we overlap the lncRNAs in TCGA and CGGA cohorts for further analyses. Finally, 23 lncRNA were identified. Using “ConsensusClusterPlus” packages (Consensus clustering can be used to perform an analysis when a negative expression value exists, which is different from nonnegative matrix factorization (NMF) clustering) (24), we explore the molecular classification and compared the survival probability of two clusters in TCGA and CGGA cohorts using Kaplan-Meier curve.

### Establishment and Validation of Pyroptosis-Related lncRNAs Prognostic Signature

The least absolute shrinkage and selection operator (LASSO) regression were performed to screen the potential pyroptosis-related lncRNAs of prognosis signature in TCGA cohort (25). We established the prognosis signature of glioma based on 13 pyroptosis-related lncRNAs that was identified using LASSO regression and multivariate cox regression. Using candidated lncRNAs, we calculated the risk score of each patient in TCGA and CGGA datasets (Risk $\text{score}=\Sigma_{i}^{13}Xi*Yi$ , *X* means coefficients, *Y* stands for FPKM value of each pyroptosis-related lncRNAs). With the assist of “Survival“ and “SurvivalROC” package, the Kaplan-Meier curves and ROC curves were drawn while “ggplot2” package works for PCA scatter plot. The “rms” package was used to construct the nomogram, which prominently predict the 1-,3-,5-year survival by evaluating various variables.

### GO Enrichment and KEGG Pathway Analysis

Using TCGA dataset, we divided study population into high-risk group and low-risk group according the median of risk score. We explored the differentially expressed genes (DGEs) between high-risk group and low-risk group with |log2 Fold Change (FC)| ≥1 and FDR< 0.05. DAVID database is a useful tool for gene annotation and functional analysis. GO categories are composed of cellular components, molecular function, and biological process. KEGG is a well-known database for systematic analysis of gene functions in biological pathways. After converting the gene symbol to entrezID, functional enrichment analysis on differentially expressed genes (DEGs) with |log2FC| ≥1 and FDR< 0.05 was performed with the assistance of “ClusterProfiler” package. The main enrichments would be showed in the diagram.

### Immune Infiltration Analysis and Construction of ceRNA Network

CIBERSORT is a computational method to measure the immune cell percentage from bulk tissue gene expression profiles. Based on the RNA-seq data of TCGA, we calculated the immune cells’ fraction between the low- and high-risk score by using the “preprocessCore” package. In order to assure the accuracy, P value is set to less than 0.05. We compared the immune infiltration status between high-risk and low-risk group.

The hypothetical targeted mRNA and miRNA was predicted in Starbase and miRTarBase. Using 1939 DGEs, 249 differentially expressed lncRNAs and 118 differentially expressed miRNA of TCGA, we described the ceRNA network using the Cytoscape software.

### Quantitative Real-Time Polymerase Chain Reaction

We selected five lncRNAs to validate the expression of pyroptosis-related lncRNAs in glioma tissue. Six non-tumor and twelve tumor samples (6 grade II and 6 grade III according to WHO grade) were obtained from patients who received surgical treatments in our hospital. This study was approved by the Ethics Committee of Xiangya Hospital, Central South University (202109928). The tissue were first stored at -80°C. Using the RNA trizol reagent, we detected the expression of several lncRNAs with the guide of manufacturer book. The relative lncRNA expression levels were measured using the 2-δδCT methods. The primers sequences for five lncRNAs were as follows: LINC00900: forward TGCCGTGGACACTGCCAGAATT, reverse: TGCGAACAGTCCAAACACGCCA; MIR155HG forward UCUUAAAGGGAAACUGAAATT, reverse: UUUCAGUUUCCCUUUAAGATT; CRNDE forward: CAAGCGAAGCACTTAACATC; reverse: CGTACCGATGCGTAGCAGAGA; TTC28.AS1 forward: ATGCTCGCTTGTGGACTGGCAA; reverse: CGGTCTCCAACCCACTTCAGG; WAC.AS1 forward: AGAATCAACAGGCTTCAAGATAGG; reverse: GCTTGATAGAGTCCCAGAGGTC;

### Statistical Analysis

All analyses herein were finished with R version 4.0.3. The Student’s T test was used to compare the gene expression levels between two groups after the normality test while the fraction of the immune cells was valued by the Mann–Whitney test. Statistical significance of survival probability in two groups (Cluster1 and Cluster 2, or low-and high-risk group) is accessed *via* Log-rank test. Subgroup analysis were also performed among different clinical parameters (age, gender, grade, survival status, subclass, and immune score). Univariate and multivariate COX regression models were performed to explore the independent prognostic value of the risk model clearly.

## Results

### Screening for Pyroptosis-Related lncRNAs

**Figure 1:**
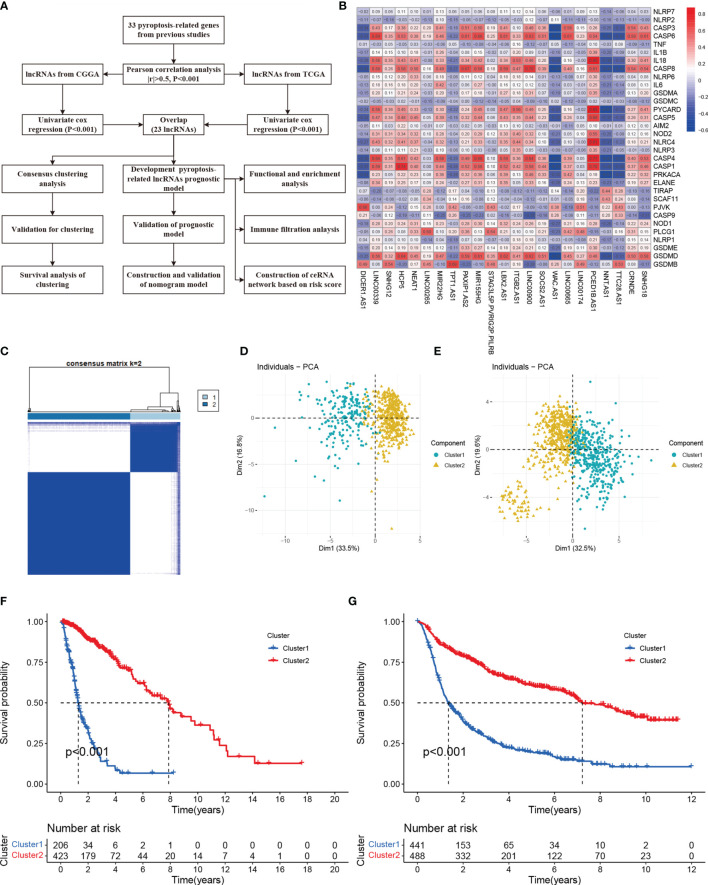
Molecular classification based on differential expressed genes. **(A)** A flow chart of study **(B)** Heatmap showed the correlation of 33 pyroptosis-related with 23 lncRNAs. **(C)** Glioma patients were divided into two clusters in TCGA. **(D)** PCA indicated that two subclasses were obtained in TCGA. **(E)** Two subclasses were validated in CGGA. **(F)** Overall survival cure of two clusters in TCGA. **(G)** Overall survival cure of two clusters in CGGA.

Combining with the GENCODE website, we annotated 14086 lncRNAs in TCGA and 1360 lncRNAs in CGGA dataset. Pearson correlation analysis with Pearson coefficient |r| > 0.5 and p < 0.001 dismiss a lot of lncRNAs. At the meanwhile, filtrating by Univariate COX analysis and intersecting in each dataset, we found 23 lncRNAs associated with prognosis. The study flow was presented in **Figure 1A**. The correlations between 23 lncRNAs and 33 pyroptosis-related genes were shown in **Figure 1B**. **Table S1** displayed the results of Univariate COX analysis.

### Molecular Classification Based on Pyroptosis-Related lncRNAs

Consensus Clustering is an algorithm used to identify cluster members and their number in datasets. Here, to acquire the best cluster numbers, we try to minimize the sampling variance by setting *k* (from 0 to 9), and pltem=0.8. As the **Figure 1C** shown, dividing all the glioma patients into two subgroups was the best choice. Principal component analysis (PCA) revealed there were distinct features between two clusters in each TCGA (**Figure 1D**) and CGGA (**Figure 1E**) dataset. Furthermore, Cluster 1 had shorter overall survival than others in the TCGA cohort (**Figure 1F**). The survival trend was also validated in the CGGA cohort (**Figure 1G**)

### Development of Pyroptosis-Related lncRNAs Prognosis Signature

**Figure 2:**
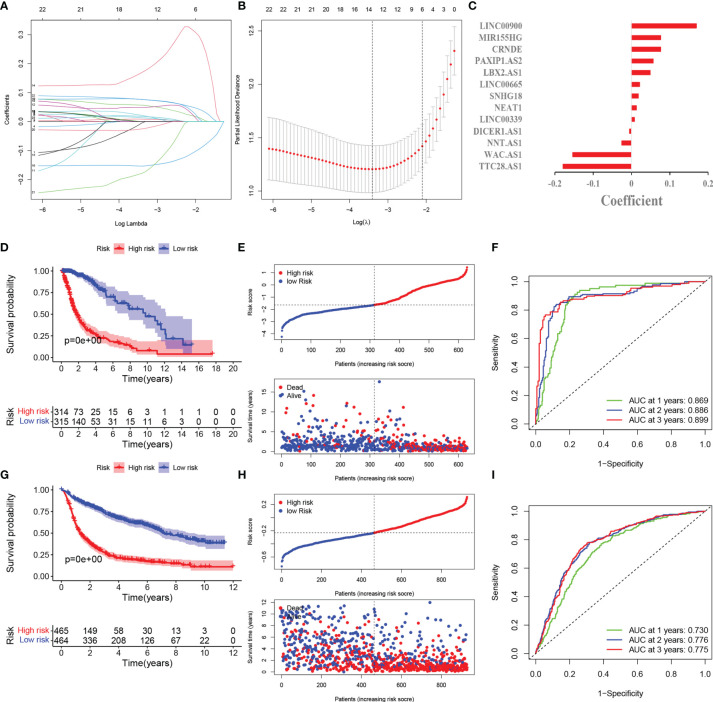
Development and validation of pyroptosis-related lncRNAs prognosis signature. **(A)** LASSO regression of 13 prognosis-related genes. **(B)** Cross-validation for tuning the parameter selection in the LASSO regression. **(C)** Coefficient of model regression. **(D)** Kaplan-Meier curves of high-risk group and low-risk group in TCGA. **(E)** Distribution of risk score and patients based on the risk score in TCGA. **(F)** ROC curves of prognostic signature based on risk score. **(G)** Kaplan-Meier curves of high-risk group and low-risk group in CGGA. **(H)** Distribution of risk score and patients based on the risk score in CGGA. **(I)** ROC curves of prognostic signature based on risk score in CGGA.

The LASSO COX regression analysis help construct a multi-lncRNA signature in the TCGA cohort. According to the λ, we identified 13 lncRNAs involved and applied to calculate the risk score (**Figures 2A, B**). The high expression of nine lncRNAs (LINC0900, MIR155HG, CRNDE, PAXIP1.AS2, LBX2.AS1, LINC00665. SNHG18, NEAT1, LINC00339) and low expression of four lncRNAs (DICER1.AS1, NNT.AS1, WAC.AS1, TTC28.AS1 were with poor prognosis in glioma (**Figure S2**). The corresponding coefficient value was shown in **Figure 2C**. Based on the median of the risk score, we divided the glioma patients into high-risk and low-risk groups. The PCA and t-SNE analysis indicated the distinct features between them (**Figure S1**).

The survival probability of high-risk group is lower than that of low-risk group (**Figure 2D**). The scatter plots showed the risk score and the survival time of each patients indicated there were more deaths and shorter survival years in high-risk group (**Figure 2E**). The AUC at 1,2,3 years are 0.869,0.886,0.899 correspondingly, which illustrated the precision of the signature is relatively high (**Figure 2F**).

### External Validation of Pyroptosis-Related lncRNAs Prognosis Signature

929 patients from CGGA database were incorporated as the validation cohort. We extracted the relevant lncRNAs and calculated the risk score as the TCGA cohort did. According to the median of the risk score in TCGA cohort, 929 patients were divided into two groups. The survival rate was longer in low-risk group and there were more deaths in high-risk group (**Figures 2G, H**). These results are consistent with the TCGA cohort. The AUC of 1,2,3 years are 0.730,0.776,0.775 respectively, which meant the model had a good predictive capability (**Figure 2I**).

**Figure 3:**
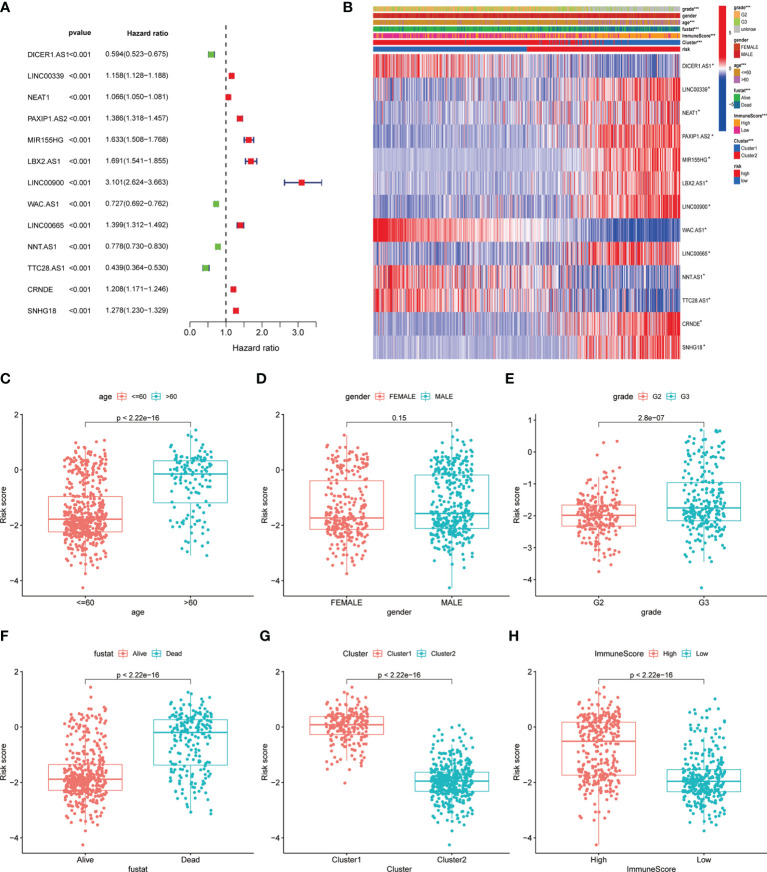
Correlation of clinical characteristic with identified pyroptosis-related lncRNAs signature. **(A)** Forest of univariate COX regression for 13 signature lncRNAs. **(B)** Heatmap showed that correlation of clinical parameters with risk score and expression of 13 lncRNAs in high- and low-risk group. Boxplot showed the comparisons of risk score in different subgroup: **(C)** age<=60 vs >60, **(D)** Female vs Male. **(E)** Grade 2 vs Grade 3. **(F)** Dead vs Alive, **(G)** Cluster 1 vs Cluster 2. **(H)** High immune score vs low immune score. *P < 0.05; **P < 0.001; ***P < 0.0001.

The univariate cox regression indicated that 13 lncRNAs were associated with poor prognosis in glioma (**Figure 3A**). The heat map including lncRNAs expression and other clinical information (grade, gender, age, survival state, immune score and cluster) revealed the expression of NNT.AS1, TTC28.AS1, WAC.AS1, DICER1.AS1 is negatively correlated with the risk score while the immune score is positively correlated (**Figure 3B**). At the meantime, we observed the patients older than 60 or belonging to the cluster 1 had a higher risk score while the gender made no significant difference (**Figures 3C–E**). However, the higher the risk score was, the more dead there were (**Figure 3F**). The cluster 1 and group with high immune score also have higher risk scores (**Figures 3G, H**).

**Figure 4:**
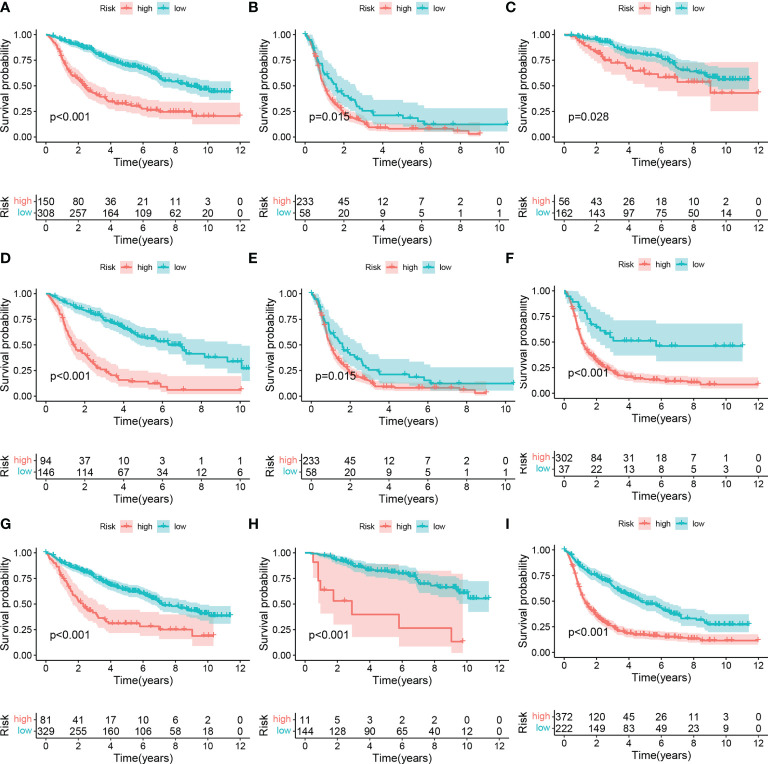
Subgroup analysis of high- and low-risk group. **(A)** LGG, **(B)** GBM. **(C)** WHO II, **(D)** WHO III, **(E)** WHO: IV. **(F)** IDH wildtype, **(G)** IDH mutant, **(H)** 1p9ql codeletion, **(I)** 1p19ql non-codeletion.

To further explore whether the risk score can be used in other situations, we performed the subgroup analysis. We found the risk score can predict the overall survival of the patients with different (**Figures 4A–I**). These outcomes pointed out the predictive potential of risk score.

### Independent Prognostic Analysis of Risk Score and Clinical Correlation

**Figure 5:**
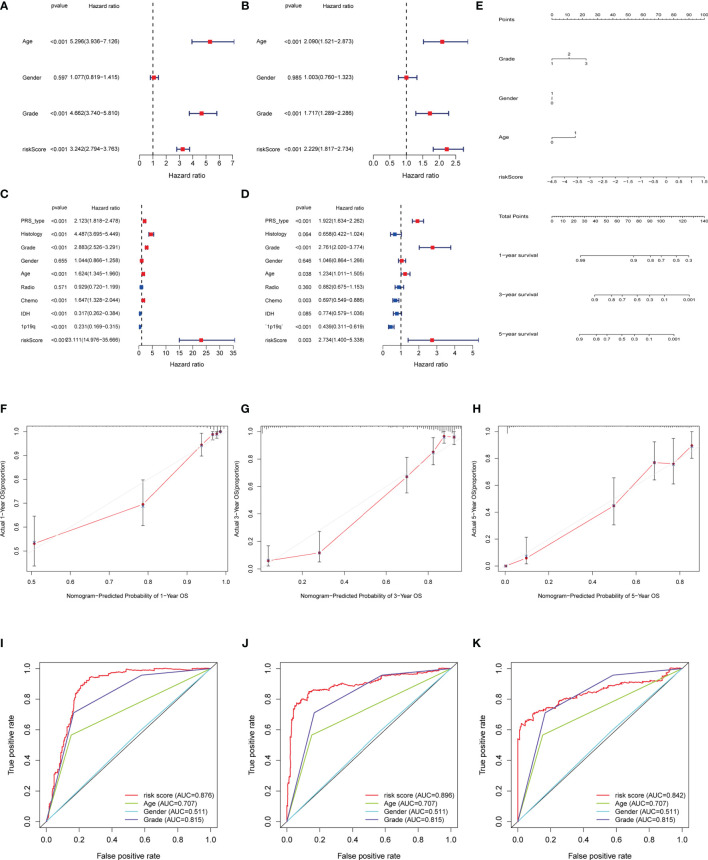
Independent prognosis analysis of risk score. **(A, C)** Univariate COX Forest plot of risk score in TCGA and in CGGA. **(B, D)** Multivariate COX Forest plot of risk score in TCGA and CGGA. **(E)** Nomograph plot of predicted 1-,3-and 5-year overall survival probability based on prognosis signature. **(F–H)** Calibration plots of the nomogram for predicting the probability of OS at 1, 3, and 5 years in the TCGA. **(I–K)** Time-dependent receiver operating characteristic (ROC) curves for the nomogram, risk score, age and grade in the TCGA dataset (for predicting 1, 3, and 5-year OS).

To construct the model that not only takes more relevant clinical information into consideration, but also facilitates clinical application, we firstly identified the independently prognostic factors of glioma patients and established the nomogram.\As the results of univariate and multivariate COX regression shown in **Figures 5A–D**, the hazard ratio of age, grade and risk score are 5.1, 2.1, 3.1, which indicated they could be the independent predictors. The nomogram could calculate the survival probability precisely based on the grade, gender, age and the risk score of the patients (**Figure 5E**). The calibration plots indicated that the observed vs predicted rate is of 1-, 3-, 5- year OS showed perfect concordance in the TCGA cohort (**Figures 5F–H**). The 1-,3-. 5-year ROC curve also validated that the predication accuracy of risk score based on pyroptosis-related lncRNAs are the highest in TCGA cohort (**Figures 5F–K**) and CGGA cohort (**Figure S3**) (**Figures 5F–K** and **Figure S3**).

### GO and KEGG Pathways Analysis

**Figure 6:**
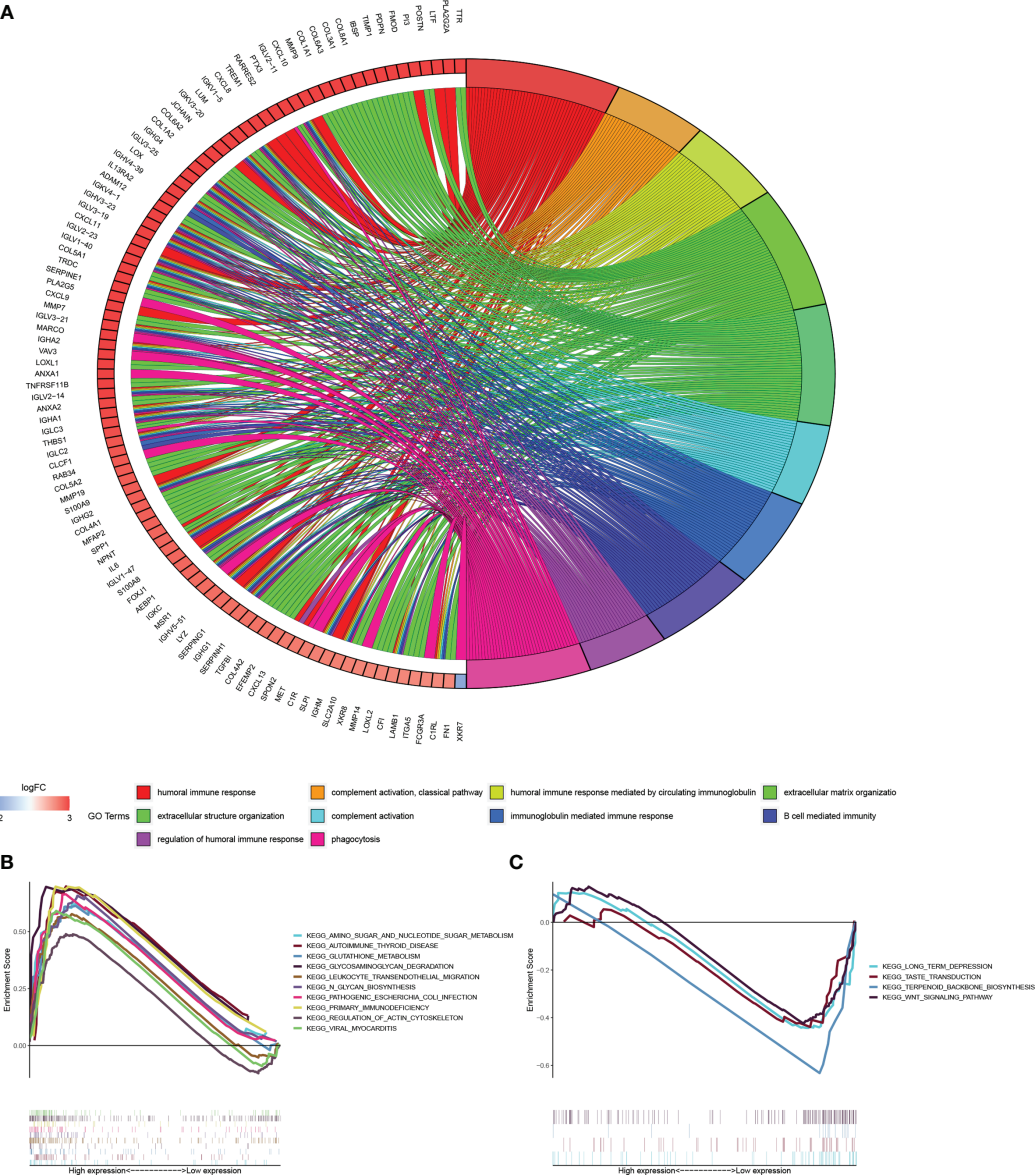
Functional and enrichment pathways analysis. **(A)** Go circle plot of enrichment analysis based on differently expressed genes between high- and low-risk groups. **(B)** KEGG pathway enrichment analysis in high-risk group. **(C)** KEGG pathway enrichment analysis in low-risk group.

According to the tendency of gene expression in the high-and low risk score group, we quantified and performed GO and KEGG pathways analysis to uncover the function and pathways. The DEGs [|log2 (fold change) | > 2 and p < 0.05] in two groups was displayed in **Table S2**. These DEGs mainly enriched in terms: humoral immune response, complement activation, humoral response mediated by circulating immunoglobulin, extracellular matrix and structure organization, B cell mediated immunity, immunoglobulin mediated immune response, regulation of humoral immune response (**Figure 6A**). Gene set enrichment analysis (GSEA) indicated the key pathways, such as, amino sugar and nucleotide sugar metabolism, autoimmune thyroid disease, glutathione metabolism, primary immunodeficiency in high-risk score group and long-term depression in low-risk score groups (**Figures 6B, C**).

### Immune Status Analysis Between Based on Risk Score

**Figure 7:**
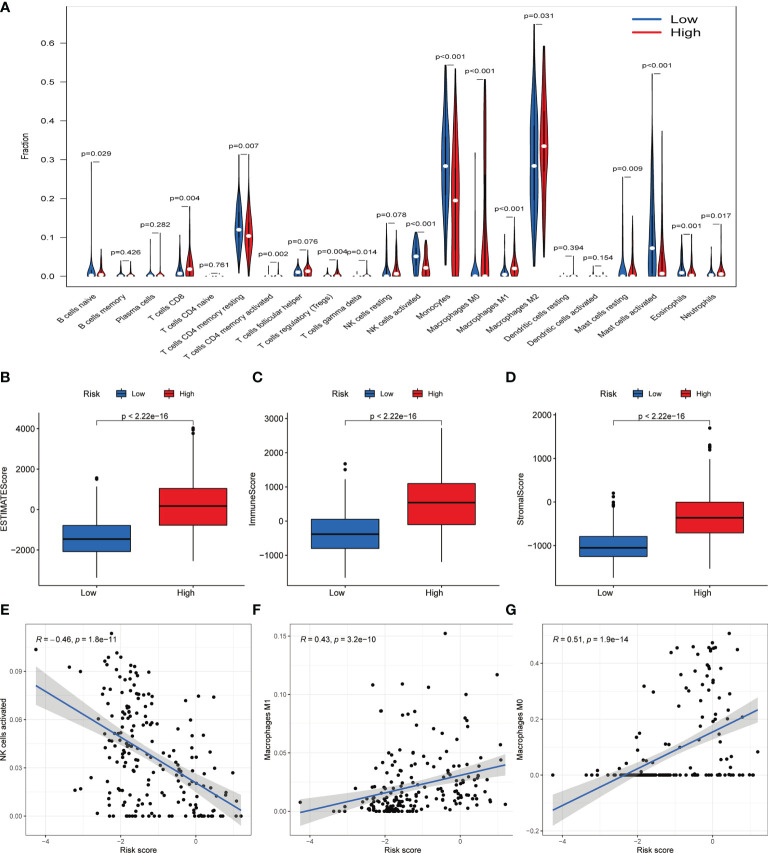
Immune filtration analysis between high- and low-risk groups. **(A)** Differential analysis of immune-related cells based on risk score. **(B–D)** Boxplot showed the comparisons of Estimation, immune and stromal score between high- and low-risk groups. **(E–G)** Scatter plot showed that the correlations of risk score with NK cells activated, Macrophages M1 and Macrophages M0.

Due to the GO analysis revealed several processes related to immunity in high-risk group, we evaluated the immune filtration, which showed immune cells such as CD8+ T cell, macrophages M2 is relatively high in high-risk group (**Figure 7A**). Meanwhile, Boxplot also presented the estimation, immune and stromal score is higher in high-risk group (**Figures 7B–D**). Further exploration found the NK cells activated is negatively correlated with the risk score while the opposite in macrophages M1 and M0 (**Figures 7E–G**).

### Construction of ceRNA Network and Functional Enrichment Analysis

**Figure 8:**
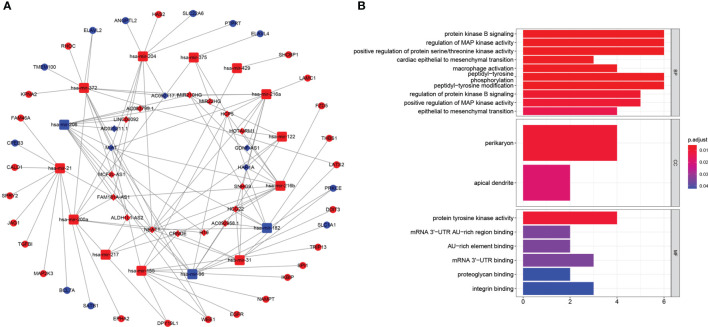
**(A)** The ceRNA network of the seven pyroptosis-related lncRNAs (rhombus) and their target miRNAs (quadratee) and mRNAs (circle). **(B)** Bar plot showed that functional enrichment of targeted mRNA in the network.

To illustrate how lncRNAs regulated the expression of targeted mRNA, we investigated the miRNA sponged by lncRNAs with the assist of TargetScan database. Finally, we identified 20 lncRNAs (upregulated: 15; downregulated: 5), 16 mir-RNA (upregulated: 13; downregulated: 3) and 35 mRNAs (upregulated: 24; downregulated: 11) for further analysis. The ceRNA network showed in **Figure 8A** clearly explained the specific regulation mechanism. GO analysis revealed targeted mRNA enriched in protein kinase B signaling, regulation of MAP kinase activity, positive regulation of protein serine/threonine kinase activity, epithelial to mesenchymal transition, peptidyl-tyrosine phosphorylation modification (**Figure 8B**).

### Validation of Expression Levels of Pyroptosis-Related lncRNAs in Glioma Samples

To validate the expression levels of pyroptosis-related lncRNAs in tumor samples, we examined the expression levels of (LINC00900, MIR155JG, CRNDE) and negative (TTC28.AS1and WAC.AS1) in 6 non-tumor tissue and 12glioma samples (6 grade II and 6 grade III). The results indicated that LINC0090, MIR155JG and CRNDE were significantly upregulated in glioma samples, and TTC28.AS1and WAC.AS1 were downregulated. Significant differences were also observed among between grade II and grade III (**Figure S4**).

## Discussion

The present study indicated that pyroptosis-related lncRNAs can be used to classify glioma patients into two subclasses based on different molecular features and clinical characteristics. The established prognostic model based on 13 pyroptosis-related lncRNAs not only predicted the prognosis of glioma patients but also reflected the molecular alterations, and immune infiltration status of different risk groups. The classification based on the risk score of prognostic signature genes revealed a lncRNA-miRNA-mRNA regulatory network. Our study provides a new understanding of pyroptosis-related lncRNAs in the development and progression of glioma.

Glioma is a common intracranial tumor with many risk factors. The extensive research on genetic alterations not only help to clarify the pathogenesis of glioma, but also predict the prognosis of patients. Previous Studies had suggested pyroptosis was associated with human inflammatory diseases and tumors, and it could develop into a therapeutic strategy (26). Chemotherapeutic drugs such as doxorubicin and topotecan (27), some natural products (28), and other reagents play an anti-tumor role by inducing pyroptosis from different pathways. LncRNA is a non-coding RNA with relatively long (> 200 nt) transcripts. It is confirmed that the lncRNAs can make a difference on pathogenicity of cancer (29, 30). It is reported that LncRNA GAS5 induces the occurrence of ovarian cancer *via* inflammasome formation during pyroptosis process (31). In contrast, some high expression of lncRNAs can inhibit cell pyroptosis (32). The downregulation of lncRNA XIST inhibits the development of NSCLC by mediating pyroptosis (14). It was also showed the knockout of lncRNA RP1-85f18.6, highly expressed in colorectal cancer, would promote pyroptosis (33). Besides, LncRNA and pyroptosis was also associated with the occurrence, development of cancers, and oxidative stress and radiosensitivity of cancers (34). Some prediction models based on autophagy gene, immune gene and ferroptosis gene have been established in gliomas. However, there is no publication elucidating the correlation of pyroptosis-related lncRNAs with glioma. Here, it is the first report that presents the role of pyroptosis-related lncRNAs in predicting overall survival and immune infiltration of gliomas. In addition, the establishment of nomogram help physicians make clinical decisions.

Here, we construct a signature of pyroptosis-related lncRNAs to predict the overall survival and the immune landscape of glioma patients. We firstly obtained the absolutely different datasets from TCGA and CGGA, and performed Pearson correlation analysis with Pearson coefficient |r| > 0.5 and P < 0.001. This is the first systematic bioinformatics analysis of pyroptosis-related lncRNAs in glioma.

How lncRNA regulated the occurrence of pyroptosis have not been thoroughly elaborated and the mechanisms mainly focus on: lncRNA binds miRNA through sponge adsorption to regulate the expression of miRNA and changes the expression levels of proteins (9, 11–13). Among these pyroptosis-related lncRNAs, the biological functions of some lncRNAs were confirmed. High expression of LncRNA SNHG18 indicated poor prognosis in multiple myeloma and hepatocellular carcinoma (35, 36). Furthermore, it also promoted the radioresistance of glioma (37). Negative correlation was found between lncRNA HCP5 and radiosensitivity of glioma (38). The function of some lncRNAs had been reported. The lncRNA SNHG12 modulated the glioma cell growth and enhanced the tumor malignancy (39, 40). LncRNA Linc00174 is not only positively related to the chemoresistance (41), but also a considerable prognosis biomarker (42, 43). LncRNA ITBG2-AS1 and lncRNA MIR155HG can be a marker for prognosis and immunotherapy in multiple cancers (44, 45).

In order to discover biological characteristics and conduct a further accurate treatment, we grouped heterogeneous patients in TCGA cohort and found that patients in cluster1 had a shorter survival time. We identified 13 pyroptosis-related lncRNAs for prognosis signature. Most of the coefficients of LncRNAs were positive, which may lead to a higher risk score and a worst survival probability. These results were consistent with the experimental outcomes of lncRNA CRNDE, lncRNA LBX-AS1, lncRNA SNGH18, lncRNA NEAT1. We tried to find the relationship between risk score and patients’ clinical information, and found that patients’ age, glioma grade and survival status, cluster and immune score were positively correlated with risk score, Moreover, the risk score calculated by our formula can identify the survival probability of patients with different clinical features including grade, IDH and 1p19q1 status. These interested us to explode the clinical application of the risk score and whether the clinical features would contribute to the survival probability. Because the recorded data was different in TCGA training cohort and CGGA validation cohort, different factors were considered when calculating the survival probability. However, nomogram here was not exactly the same as previous studies (22, 46).

In our analysis, several pathways were enriched in high-risk score group, such as humoral immune response, complement activation, classical pathway and immunoglobulin mediated immune response, B cell mediated immunity, extracellular structure and matrix organization and so on. Extracellular matrix was an important part of tumor microenvironment, and its components and interaction with glioma cell would make different on the malignant transformation, especially the infiltration and invasion ability of glioma (47, 48). It was gratifying that extracellular matrix could act as the target of drugs, which might be one of the methods to treat glioma (49). KEGG enrichment revealed the process of amino sugar and nucleotide sugar metabolism, glutathione metabolism. As we all know, alterations metabolism of cancer might affected the biological processes of cells contributing to the development and progression (50). Focusing on the Warburg effect also assisted the management of glioma (51). It is more striking that immunotherapy for glioma is a hot spot at present because of its ability to penetrate the blood-brain barrier (52). The pathways related to the immunity found in our results urged us evaluate the immune filtration of patients. Macrophages M2, monocytes and resting CD4+ T cells made up a large part of the immune cells. Furthermore, the fact that different cells have distinct correlations with risk score (positive or negative correlation) called our attention to different treatment strategies for different groups. Finally, we constructed the ceRNA network to show the most possible and most common mechanisms of the lncRNAs in glioma clearly.

Although the main problem that performs a model to value the prognosis and the immune landscape robustly, there are some limitations in our study. First, replacing the age with diagnosis age in nomogram might more accuracy in evaluating the survival probability. Second, it is necessary to expand the sample size to verify and modify the model repeatedly. Third, pyroptosis is a newly discovered form of cell death, so there are restricted researches elucidating its relationship with lncRNAs in glioma. More studies are required on whether lncRNAs we found here are related to pyroptosis and the specific mechanism of inducing pyroptosis.

In short, recognizing the limited predictive capability of single lncRNA, we screened for pyroptosis-related lncRNAs and modeled it in gliomas. This model can predict the survival rate of glioma patients by detecting the expression of a few lncRNAs and combining with the clinical information of patients. More significantly, this model focusing on the direction of pyroptosis, provides new ideas for finding new therapeutic methods for gliomas.

## Data Availability Statement

The datasets presented in this study can be found in online repositories. The names of the repository/repositories and accession number(s) can be found in the article/**Supplementary Material**.

## Ethics Statement

The studies involving human participants were reviewed and approved by Ethics Committee of Xiangya Hospital, Central South University. The patients/participants provided their written informed consent to participate in this study.

## Author Contributions

ZL designed this study and contributed substantially to the design of the search strategy. GT searched and selected the trials and extracted data. ZL performed the analysis and interpreted the data. GT wrote the manuscript. ZL critically reviewed the manuscript. NL participated in the data extraction and critically revised it. ZL, LS, and RZ proofread the final version. All authors read and approved the final manuscript.

## Funding

This study was supported by the National Natural Science Foundation of China (No. 82003239), Hunan Province Natural Science Foundation (Youth Foundation Project) (NO.2019JJ50945), and the Science Foundation of Xiangya Hospital for Young Scholar (NO. 2018Q012).

## Conflict of Interest

The authors declare that the research was conducted in the absence of any commercial or financial relationships that could be construed as a potential conflict of interest.

## Publisher’s Note

All claims expressed in this article are solely those of the authors and do not necessarily represent those of their affiliated organizations, or those of the publisher, the editors and the reviewers. Any product that may be evaluated in this article, or claim that may be made by its manufacturer, is not guaranteed or endorsed by the publisher.

## Supplementary Material

Supplementary Figure 1
**(A)** PCA analysis showed the distribution of two risk subclasses in the TCGA dataset **(B)** t-SNE analysis supported the stratification into two risk groups in TCGA. **(C)** PCA analysis showed the distribution of two risk subclasses in the CGGA **(D)** t-SNE analysis supported the stratification into two risk groups in CGGA

Supplementary Figure 2 Kaplan-Meier curves of 13 identified pyroptosis-related lncRNAs in TCGA. **(A–M)** CRNDE, DICER1.AS1, LBX2.AS1, LINC00339, LINC00665, LINC00900, MIR155HG, NEAT1, NNT.AS1, PAXIP1.AS2, SNHG18, TTC28.AS1, WAC.AS1

Supplementary Figure 3
**(A)** Nomograph plot of predicted 1-,3-and 5-year overall survival probability based on prognosis signature. **(B–D)** Calibration plots of the nomogram for predicting the probability of OS at 1, 3, and 5 years in the CGGA. **(E–G)** Time-dependent receiver operating characteristic (ROC) curves for the nomogram, risk score, age and grade in the CGGA dataset (for predicting 1, 3, and 5-year OS).

Supplementary Figure 4 Relative expression of pyroptosis-related lncRNAs in tumor samples. **(A)** LINC00900; **(B)** MIR155HG; **(C)** CRNDE; **(D)** TTC28.AS1; **(E)** WAC.AS1
