# Supplementary figures and images for: Molecular Subtypes and Prognostic Signature of Pyroptosis-Related lncRNAs in Glioma Patients
Source: Front Oncol. 2022 Feb 14;12:779168. doi: 10.3389/fonc.2022.779168 (PMC8884250; doi:10.3389/fonc.2022.779168)

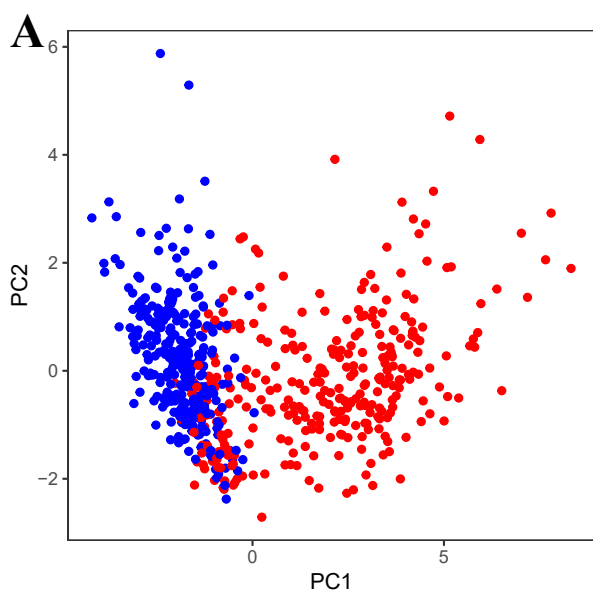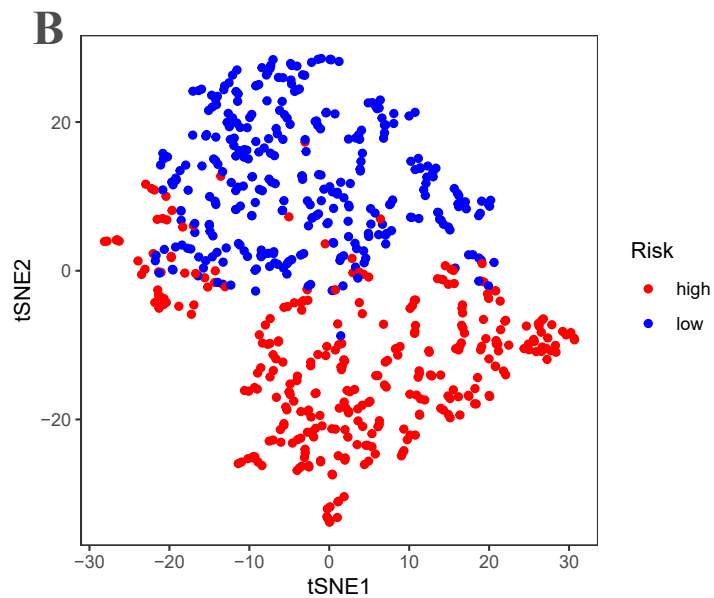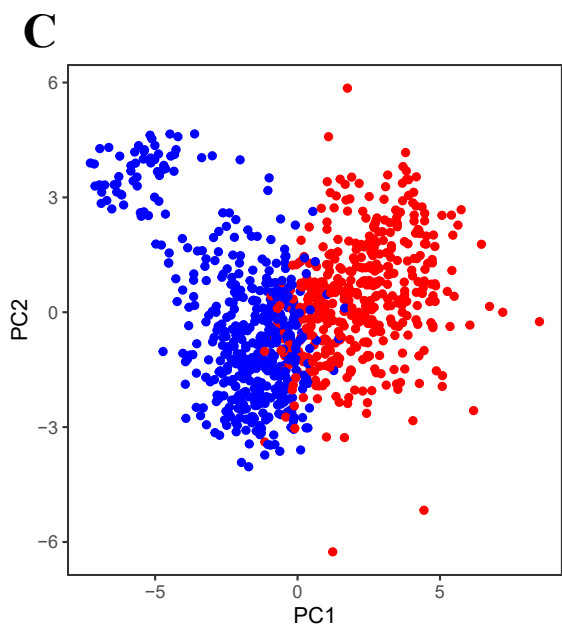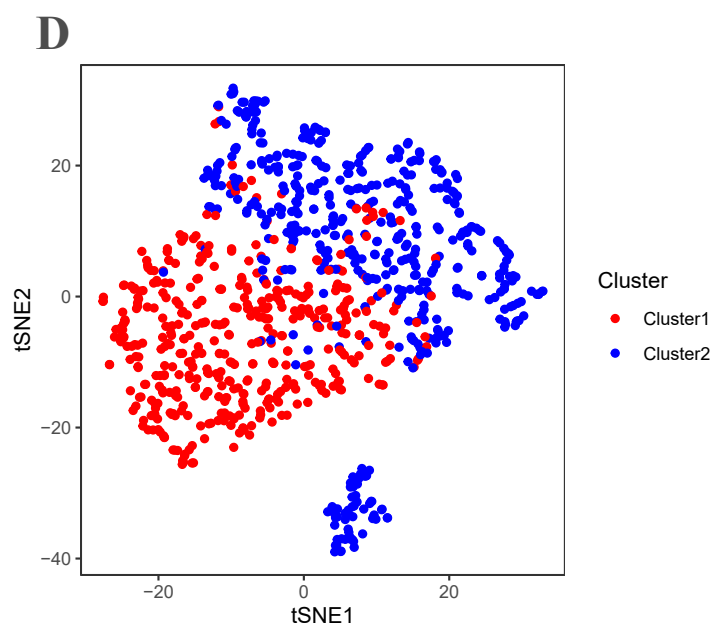

Supplement: Supplementary Figure 1 — (A) PCA analysis showed the distribution of two risk subclasses in the TCGA dataset (B) t-SNE analysis supported the stratification into two risk groups in TCGA. (C) PCA analysis showed the distribution of two risk subclasses in the CGGA (D) t-SNE analysis supported the stratification into two risk groups in CGGA [file DataSheet_1.pdf]

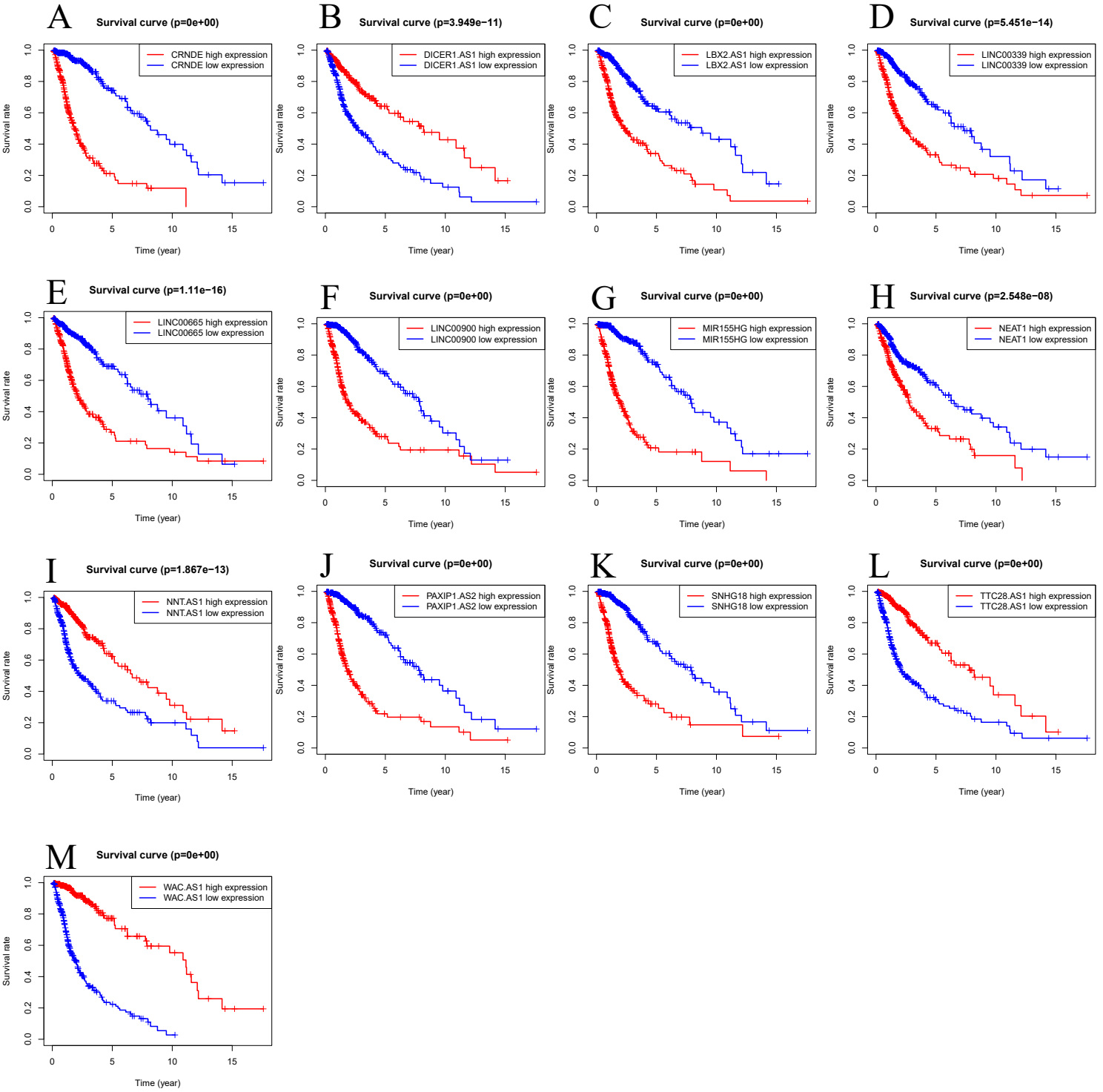

Supplement: Supplementary Figure 2 — Kaplan-Meier curves of 13 identified pyroptosis-related lncRNAs in TCGA. (A–M) CRNDE, DICER1.AS1, LBX2.AS1, LINC00339, LINC00665, LINC00900, MIR155HG, NEAT1, NNT.AS1, PAXIP1.AS2, SNHG18, TTC28.AS1, WAC.AS1 [file DataSheet_2.pdf]

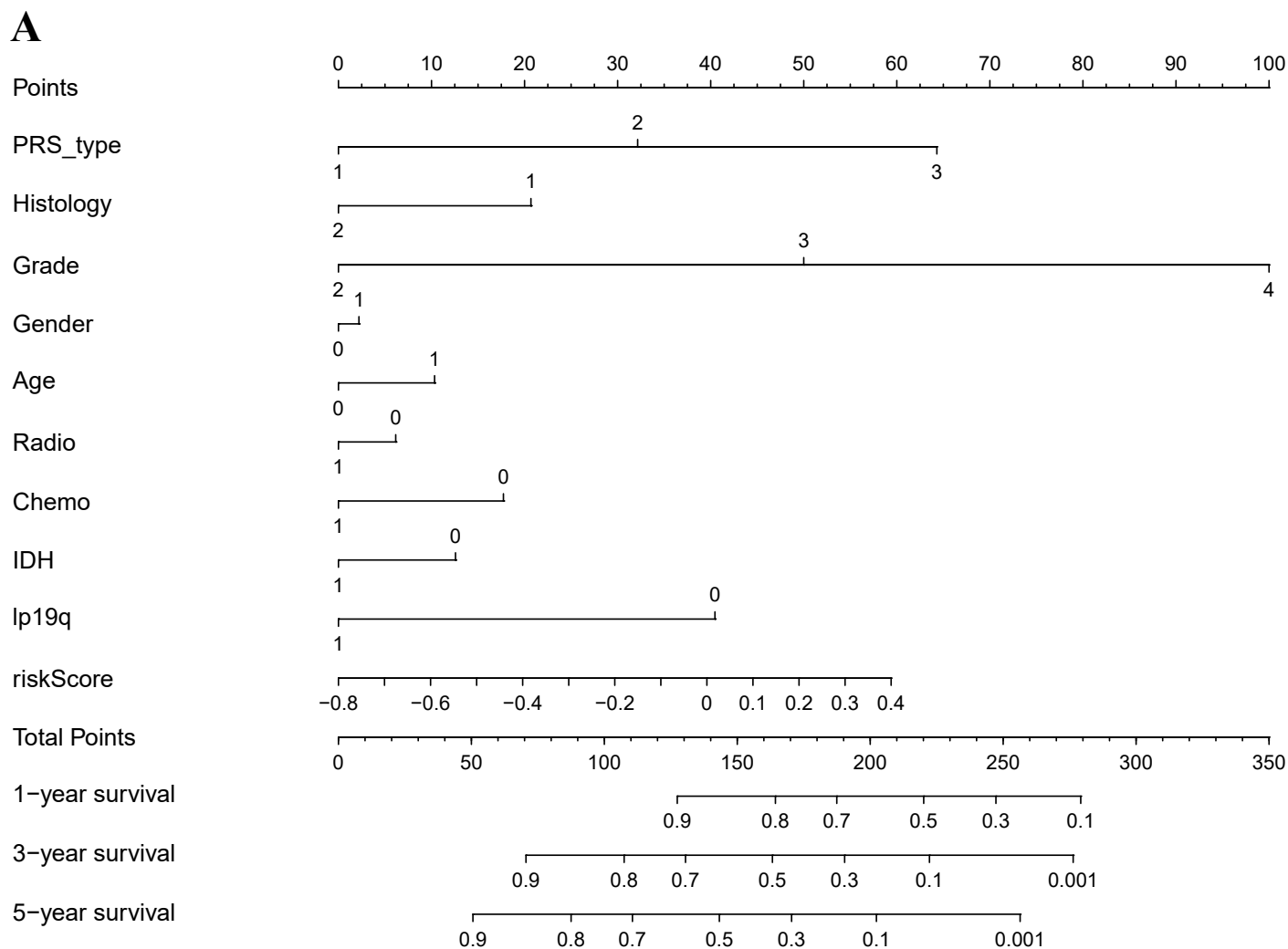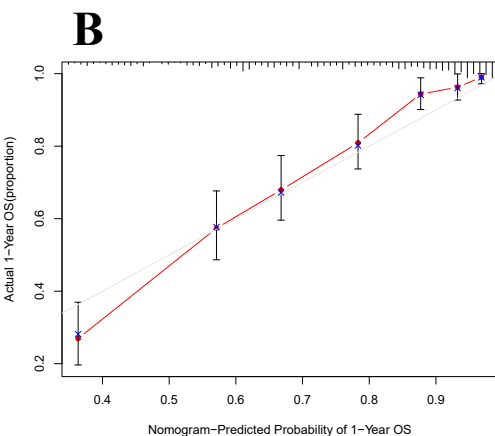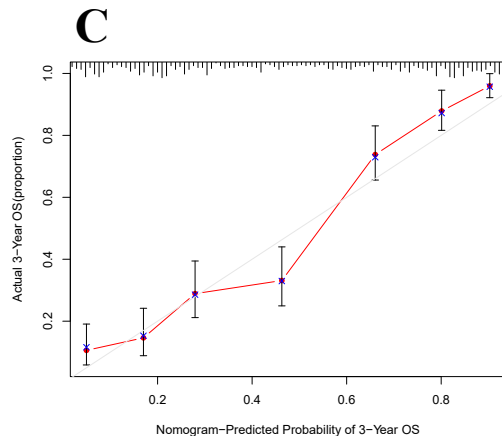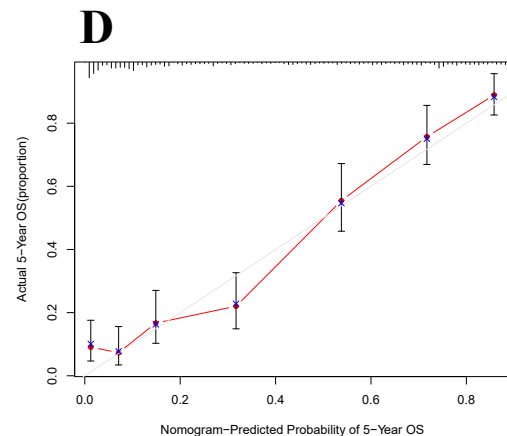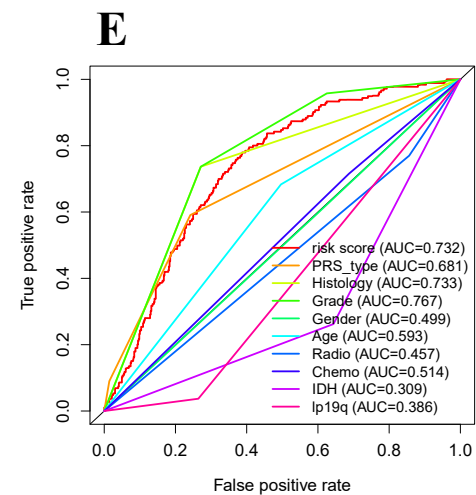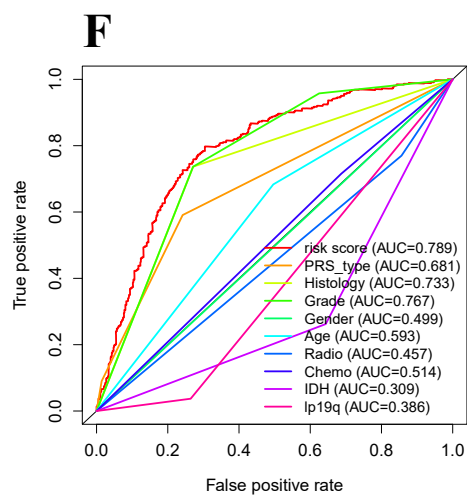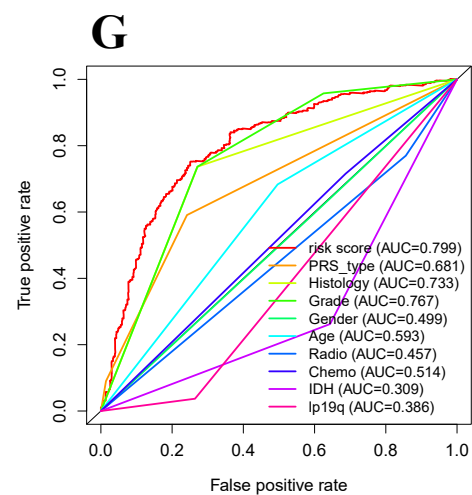

Supplement: Supplementary Figure 3 — (A) Nomograph plot of predicted 1-,3-and 5-year overall survival probability based on prognosis signature. (B–D) Calibration plots of the nomogram for predicting the probability of OS at 1, 3, and 5 years in the CGGA. (E–G) Time-dependent receiver operating characteristic (ROC) curves for the nomogram, risk score, age and grade in the CGGA dataset (for predicting 1, 3, and 5-year OS). [file DataSheet_3.pdf]

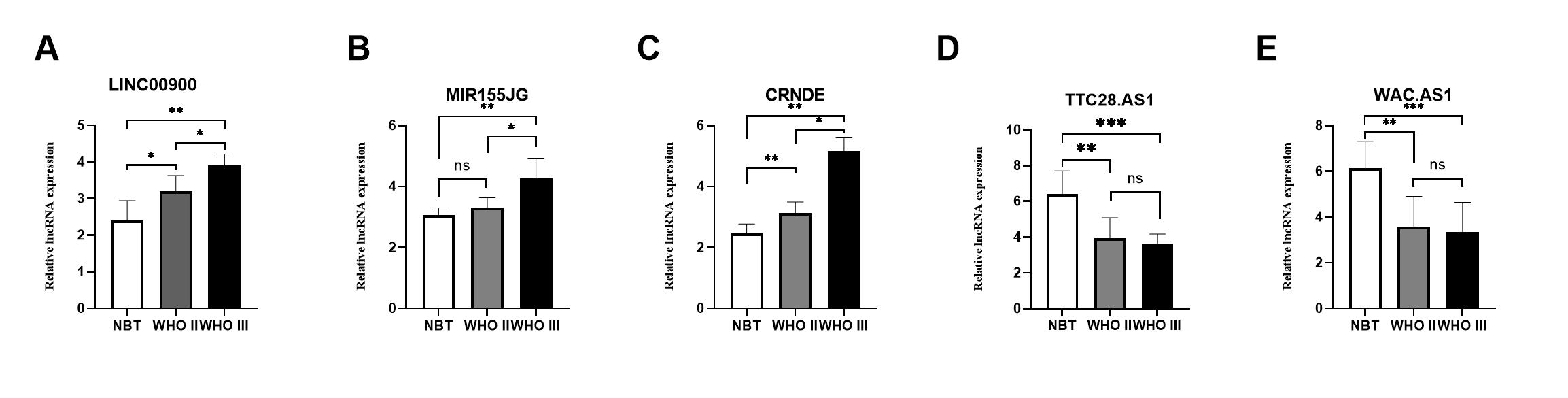

Supplement: Supplementary Figure 4 — Relative expression of pyroptosis-related lncRNAs in tumor samples. (A) LINC00900; (B) MIR155HG; (C) CRNDE; (D) TTC28.AS1; (E) WAC.AS1 [file Image_1.tif]
